# Prediction of Second Language Proficiency Based on Electroencephalographic Signals Measured While Listening to Natural Speech

**DOI:** 10.3389/fnhum.2021.665809

**Published:** 2021-07-16

**Authors:** Aya S. Ihara, Atsushi Matsumoto, Shiro Ojima, Jun’ichi Katayama, Keita Nakamura, Yusuke Yokota, Hiroki Watanabe, Yasushi Naruse

**Affiliations:** ^1^National Institute of Information and Communications Technology, and Osaka University, Kobe, Japan; ^2^Department of English, College of Education, Yokohama National University, Yokohama, Japan; ^3^Department of Psychological Science, and Center for Applied Psychological Science (CAPS), Kwansei Gakuin University, Nishinomiya, Japan; ^4^Eiken Foundation of Japan, Shinjuku, Japan

**Keywords:** EEG, language proficiency, speech, foreign language, second language, multivariate temporal response function

## Abstract

This study had two goals: to clarify the relationship between electroencephalographic (EEG) features estimated while non-native speakers listened to a second language (L2) and their proficiency in L2 determined by a conventional paper test and to provide a predictive model for L2 proficiency based on EEG features. We measured EEG signals from 205 native Japanese speakers, who varied widely in English proficiency while they listened to natural speech in English. Following the EEG measurement, they completed a conventional English listening test for Japanese speakers. We estimated multivariate temporal response functions separately for word class, speech rate, word position, and parts of speech. We found significant negative correlations between listening score and 17 EEG features, which included peak latency of early components (corresponding to N1 and P2) for both open and closed class words and peak latency and amplitude of a late component (corresponding to N400) for open class words. On the basis of the EEG features, we generated a predictive model for Japanese speakers’ English listening proficiency. The correlation coefficient between the true and predicted listening scores was 0.51. Our results suggest that L2 or foreign language ability can be assessed using neural signatures measured while listening to natural speech, without the need of a conventional paper test.

## Introduction

Second language (L2) education is becoming increasingly important in the era of globalization. English has become a lingua franca for speakers of different native languages, and English language education has spread to many parts of the modernized world. One important aspect of L2 education is the method of assessment. Currently, several major tests of English as an L2, such as Test of English as a Foreign Language, are available for assessing English proficiency. Taking advantage of sophisticated statistical theories, such as Item Response Theory (Wainer et al., 2000), these tests are able to reliably assess L2 proficiency.

However, conventional L2 tests treat language processing as a “black box” and use behavioral responses generated after language processing as the basis of assessment. For example, in a typical listening comprehension test, one listens to L2 sentences and is required to choose the most appropriate answer from multiple choices. The test score is determined by the answers chosen, not by the actual mental processes that occurred while the sentences were being processed. When the answer is wrong, something must have gone wrong in the process of listening comprehension, but that usually remains unknown in conventional tests.

Brain imaging allows us to obtain detailed information about language processing. Advances in brain imaging technology have made it possible to visualize language processing in the human brain (Poeppel, 2014). Event-related brain potentials (ERPs), in particular, can reveal the millisecond-by-millisecond time courses of neural activity involved in language processing as it unfolds in the brain (Luck, 2014).

Past ERP studies have suggested that the possibility that L2 ability can be assessed by neural signatures alone. A rich body of research has shown that proficiency in an L2 is one of the major factors that determine brain responses to stimuli in that language (for review, Caffarra et al., 2015). For example, ERPs recorded from L2 learners vary as a function of L2 proficiency. Syntax-related ERP components such as the P600 are often missing or reduced in size in low-proficiency L2 learners. The N400 component, which is modulated by semantic factors, is usually present even in low-proficiency learners, but their N400 latencies may be later than those of high-proficiency learners (Ojima et al., 2005). Overall, an increase in L2 proficiency leads to systematic changes in neural signatures (Kotz, 2009), which could be used systematicity for the assessment of L2 proficiency.

To achieve this goal, we must first address the problems associated with the ecological validity of brain imaging studies of language. In most neuroimaging experiments, linguistic stimuli are presented in a unit-by-unit manner. For example, ERP studies of sentence processing in the visual modality commonly present words in the center of the display, one word at a time (Kutas and Hillyard, 1980). However, such a mode of reading is rarely experienced in daily life. Functional magnetic resonance imaging studies and auditory ERP studies are less artificial because they usually present one whole sentence at a time (Friederici et al., 1993; Embick et al., 2000); yet, even so, everyday language involves reading or listening to a series of sentences continuously. To tackle these problems, our present study not only chose the auditory presentation mode but also used natural conversations and monologs as stimuli, each of which consisted of a series of sentences representing a cohesive story.

In addition, a significant proportion of the sentence stimuli in these experiments contained some form of violation, such as syntactic errors or semantic incongruencies (e.g., Neville et al., 1991; Friederici et al., 1993). This violation paradigm is useful for probing a particular aspect of linguistic processing, such as syntax and semantics. In this paradigm, approximately 25–50% of the sentence stimuli contain a violation to ensure a sufficiently high signal-to-noise ratio for the violation condition. This leads to a further reduction in ecological validity because the rate of anomalies (e.g., inadvertent grammatical mistakes or slips of the tongue) is far lower in typical language use; such phrase structure violations commonly used in these paradigms are rarely encountered in everyday language.

Another important problem with previous studies concerns sample size. In a typical ERP study of L2 processing, the experimental group consists of approximately 10–20 participants (Caffarra et al., 2015); studies with >50 participants in total are uncommon. The use of brain imaging for the assessment of L2 ability requires imaging data from L2 learners with a wide range of proficiency. Results from different studies cannot be combined to achieve this range because different experimental settings likely produce different results, even in the same set of participants. This necessitates that L2 learners with a broad range of proficiency levels be tested in a single study that uses consistent experimental parameters.

Our study aimed to identify electroencephalographic (EEG) indices that show a relationship with L2 proficiency and to develop a predictive model for L2 proficiency based on these EEG indices. We measured EEG signals from >200 native Japanese speakers with a wide range of English proficiency. To ensure high ecological validity, we presented natural speech without artificial interruptions within or between sentences that mimicked a situation of normal speech listening. Furthermore, the speech stimuli did not contain any violations, such as syntactic errors or semantic incongruencies; they consisted purely of well-formed and meaningful sentences. For analysis of cortical speech processing, we applied a multivariate temporal response function (mTRF) approach. The mTRF can be thought of as a filter that describes the linear transformation of ongoing multivariate stimuli to ongoing neural response (Crosse et al., 2016). This makes it possible to separate the overlapping responses caused by different, temporally close features. Therefore, this approach is ideal to analyze EEG signals for natural speech. In fact, this approach has been used in several studies on lower-order processing, such as speech envelopes and phonemes, and higher-order processing, such as semantic processing during listening to natural speech. (Di Liberto et al., 2015; Broderick et al., 2018; Etard and Reichenbach, 2019; Paul et al., 2020). In the present study, we targeted word processing during listening to natural speech. First, we estimated mTRF separately for word class (open or closed), because previous ERP studies have shown a differential characteristic of ERPs between open class words and closed class words (closed class words elicit a slow negative shift with an onset of 400 ms; Brown et al., 1999; Munte et al., 2001). In addition, we estimated mTRF separately for word position, speech rate, and parts of speech. We then investigated whether the latencies and amplitudes of the TRF components were related to the participants’ English listening comprehension scores for Japanese speakers, conducted after the ERP experiment. Finally, we generated a predictive model for Japanese speakers’ English listening proficiency based on the EEG indices.

## Materials and Methods

### Participants

Two-hundred and five adult monolingual native Japanese speakers (98 females) who learned English as their first foreign language participated in the experiment (age range 20–39 years). All participants had normal or corrected-to-normal vision and normal hearing. They had no history of neurological or psychiatric disease. The data of six participants were excluded from the analysis because their EEG data contained large artifacts. In the remaining 199 participants (96 males and 103 females; 24.2 ± 4.8 years old), the learning experience of English was as follows: 140 started learning English at age 12 years when English education becomes compulsory, 49 began learning English between the ages of 6 and 11 years, and the remaining 10 began at age 0–5 years. Handedness for the participants was as follows: 192 right-handed [laterality quotient (LQ) ≥ 70 in 181 participants] and 7 left-handed (LQ ≤ −70 in 3 participants) as confirmed by the Edinburgh Handedness Inventory (Oldfield, 1971). The study was approved by the Ethics Committee for Human and Animal Research of the National Institute of Information and Communications Technology, Japan. All participants provided informed consent to participate in the study.

### Materials

We selected 33 samples of English speech, 17 conversations and 16 monologs from the listening tests of EIKEN (EIKEN Foundation of Japan, https://www.eiken.or.jp/eiken/en/), which is one of the most widely used English-language testing programs in Japan. The grades of speech were as follows: six conversations (mean length: 16.3 ± 1.1 s) and six monologs (18.0 ± 1.4 s) from Grade 3, which corresponds to level A1 of the Common European Framework of Reference for Languages (CEFR)^1^; four conversations (30.0 ± 1.9 s) and four monologs (30.4 ± 4.2 s) from Grade Pre-2, which corresponds to CEFR level A2; four conversations (30.8 ± 2.3 s) and four monologs (32.6 ± 3.1 s) from Grade 2, which corresponds to CEFR level B1; and three conversations (35.9 ± 1.2 s) and two monologs (71.0 ± 1.0 s) from Grade Pre-1, which corresponds to CEFR level B2. The numbers of speech stimuli for each grade were determined so that the numbers of words would be roughly the same across grades. The topics of the speech stimuli ranged widely, such as work, daily life, nature, science, travel, and history.

**FIGURE 1:**
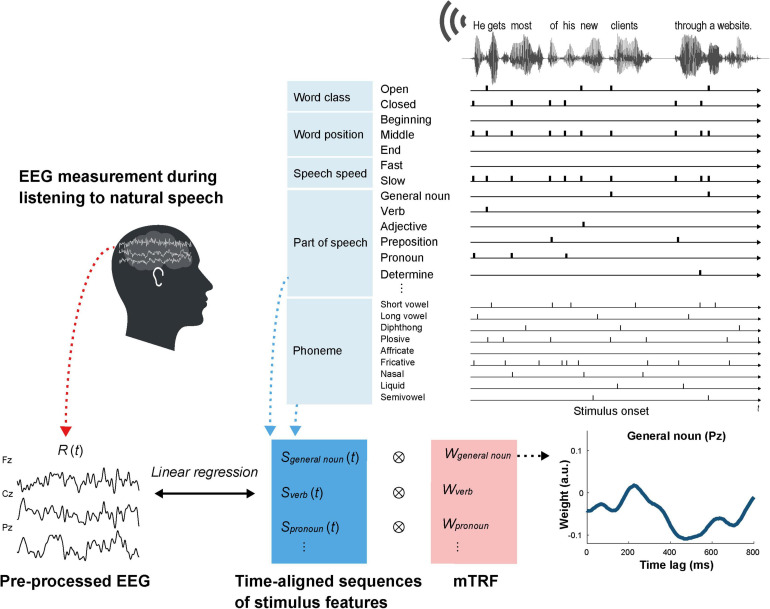
Outline of mTRF estimation for the word features in English speech.

We focused on EEG responses to the following stimulus features to investigate the relationship between brain activity and English proficiency: word class (open or closed), parts of speech, speech rate, and word position. Parts of speech for all words were identified with Text Inspector^2^, a web tool for analyzing texts. We then divided the parts of speech into the following 15 classes: general noun, proper noun, verb, adjective, adverb, and number, which are referred to as open class words; article/determiner, pronoun, interrogative word (wh-word), relative pronoun, BE verb, auxiliary verb, preposition, and conjunction, which are referred to as closed class words; and others (Supplementary Table 1). The open and closed class words were further divided into three classes based on their positions within each speech stimulus (i.e., beginning, middle, and end positions), except for interrogative sentences that were presented at the end of the speech stimuli. Speech rates for each word were determined as syllable per minute (SPM), which was calculated for each sentence containing the word. On the basis of a previous study that reported that the mean SPM rate for adult native speakers of American English is 250 ± 25 (standard deviation, SD; Robb et al., 2004), the open and closed class words were divided into two classes based on their speech rates: fast (SPM ≥ 230) and slow (SPM < 230). In addition, all phonemes were identified using Penn Phonetics Lab Forced Aligner for English^3^, an automatic phonetic alignment toolkit. The phonemes were classified by their articulatory properties: short vowel, long vowel, diphthong, plosive, affricate, fricative, nasal, liquid, and semivowel (Supplementary Table 2). A native English speaker (American man) identified the onset time for each word and phoneme and the onset and offset times for each sentence by listening to the speech stimuli and checking the sound waveforms and spectrograms using WaveSurfer^4^, an open-source speech/sound analysis tool (Figure 1).

### EEG Experiment

The experiment was divided into four blocks. The first and third blocks consisted of nine and eight conversations, respectively, and the second and fourth blocks each consisted of eight monologs. In each block, the grade of speech increased in order from Grade 3 (easy) to Grade Pre-1 (difficult). The stimulus sequence of each trial was as follows. First, the instruction “Press” was visually presented on a liquid crystal display (EIZO Corporation, Japan) that was placed in front of the participant. Each speech stimulus started with the participant pressing a key, and stimuli were presented binaurally through earphones (RHA Technologies Ltd., United Kingdom). The participants were instructed to fixate on a fixation point (+) presented in the center of the display while listening to the speech stimuli. Each conversation/monolog was followed by only one question (e.g., “How many people were at the meeting yesterday?” or “Why is Mr. Carson disappointed in Beth?”). Following the question, four choices were presented on the display. The participants were instructed to press one of the keys (1–4) that corresponded to their answer or to press 0 if they did not understand the speech stimuli. We calculated the accuracy rate for the questions in the EEG experiment for each participant. The visual and audio stimuli were presented using the Presentation stimulus delivery software (Neurobehavioral Systems, Inc., United States).

Electroencephalographic and electrooculogram (EOG) signals were continuously measured throughout all blocks using an eight-channel wireless EEG device and measurement software (Polymate Mini AP108 and Mobile Acquisition Monitor 2.02, Miyuki Giken Co. Ltd., Japan). Active electrodes were placed on Fz, Cz, Pz, FC5, and FC6 locations according to the International 10–10 system for EEG measurement, on the lateral of and above the left outer canthus for EOG measurement, and on the left earlobe for re-reference. All signals were sampled at 500 Hz using the forehead as the ground and the right earlobe as the online reference. Audio stimuli were presented stereophonically; one channel contained the speech stimuli to be presented to the participants, and the other contained a square wave as a trigger to indicate the onset of each speech stimulus, which was input into the EEG device to synchronize the EEG and EOG signals with the speech stimuli.

### English Listening Scores

After the EEG experiment, participants completed the EIKEN Institution Based Assessment (EIKEN Foundation of Japan, Japan) English listening test for Japanese speakers for approximately 20 min. The test scores (EIKEN CSE listening scores) correspond to CEFR levels as follows: <429 corresponds to level A1, 430–502 to A2, 503–602 to B1, 603–689 to B2, and 690–720 to C1 or higher^5^.

### EEG Analysis

Preprocessing of the EEG data was carried out using MATLAB (MathWorks Inc., United States) and the EEGLAB toolbox (Delorme and Makeig, 2004). All recorded signals were re-referenced to the average of both earlobes offline. After applying a bandpass FIR filter between 0.5 and 50 Hz (3,300th order), the signals were resampled at 200 Hz. Artifact subspace reconstruction was performed to remove transient, large-amplitude artifacts from the EEG data^6^. We then applied independent component analysis to the data, and artifactual components caused by eye movements and blinks were removed. Finally, we applied a low-pass FIR filter of 7 Hz (1,320th order) to the EEG data.

For each speech stimulus, we prepared four kinds of stimulus matrices (features × points) at the same sampling rate as the preprocessed EEG data: (1) word class (open and closed), (2) word position (beginning, middle, and end), (3) speech rate (fast and slow), and (4) parts of speech (Figure 1). 9 types of phonemes classified by the articulatory properties and sentence beginnings and endings were also added to each stimulus matrix. Each stimulus matrix consisted of time-aligned impulses with a value of 1 at the onset time for each feature and 0 at the other times. We used the mTRF toolbox in MATLAB (Crosse et al., 2016) to compute mTRFs that describe the linear mapping between stimulus features and the preprocessed EEG data for each electrode using each stimulus matrix. The continuous EEG response was assumed to consist of convolutions of the stimulus vectors and TRFs:

(1) $$\begin{array}{c} R_{\mathrm{ch}}\left(t\right)=\sum_{i=1}^{N^{c}}w_{i}^{c}⊗S_{i}^{c}\left(t\right)+\varepsilon_{ch}\left(t\right), \end{array}$$

where *R*_*ch*_(*t*) is the continuous EEG response at time point *t* of the *ch*th channel, *S_*i*_^*C*^*(*t*) is the stimulus vector of the *i*th feature in the *c*th stimulus matrix, *w_*i*_^*C*^* is the TRF of the *i*th feature in the *c*th stimulus matrix, *N*^*C*^ is the number of the features in the *c*th stimulus matrix, and *ε*_*ch*_(*t*) is the residual response in the *ch*-th channel. The TRF weights over the range of time lags from −200 to 1,500 ms, relative to the onset of each feature, were estimated using ridge regression with a regularization parameter, λ, of 10^2^. Optimization of the regularization parameter was performed using leave-one-out cross-validation (LOOCV) employing the mTRFcrossval function (Crosse et al., 2016). For each participant, TRFs for each channel were calculated for every ridge parameter (λ = 1, 10, 10^2^, 10^3^, and 10^4^) for each trial. Then, the trials were rotated 33 times, such that each trial was used as the test data and the remaining trials were treated as the training data. For each channel, averaged TRFs over single-trial TRFs for the training set were used to predict the neural response for the test trial; this was repeated for each of the different ridge parameters. This process was performed repeatedly until all trials were applied to the test data of each participant. Mean square errors (MSEs) between the actual and predicted responses were averaged over trials, channels, and participants (Paul et al., 2020). Finally, we obtained the optimal ridge parameter that showed the lowest MSE. We estimated the TRFs separately for word class, word position, speech rate, and parts of speech.

**FIGURE 2:**
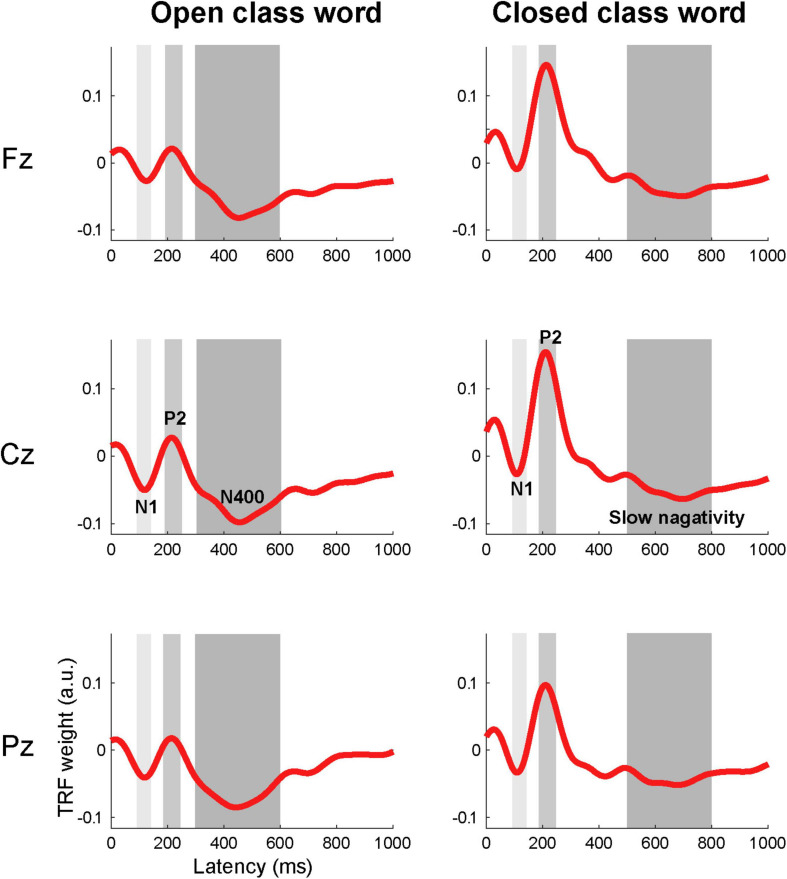
Grand-averaged TRFs across all 199 participants for open and closed class words. Two early components, corresponding to N1 and P2, are shown in the TRFs for both open and closed words at all three channels. The peak latencies were slightly shorter in the TRFs for the closed class words (N1: 110 ms; P2: 210 ms at Cz) than those for the open class words (N1: 120 ms; P2: 215 ms at Cz). The TRFs for the open class words showed a late component, corresponding to the N400, and the TRFs for the closed class words showed a later and slower negativity. The peak latencies for the open class words and the closed class words at Cz were 455 and 700 ms, respectively. Gray shades show the latency ranges used to estimate the mean amplitudes of each component: 90–140 ms for the N1 (light gray), 190–250 ms for the P2 (median gray), and 300–600 and 500–800 ms for the late component for the open and closed words (dark gray).

The TRFs were averaged across all 199 participants for each feature and identified three components: the N1, P2, and a late component (i.e., N400 for open class words and slow negativity for closed class words). We used the amplitudes and peak latencies of the three components for each feature and electrode as potential indices for predicting English proficiency. We determined the latency ranges to estimate the mean amplitudes of each component based on the grand-averaged TRF waveforms for the open and closed class words (Figure 2). For the N1 and P2 components, the amplitude of each TRF for each electrode was averaged within a latency range of 90–140 ms and 190–250 ms, respectively; these latency ranges are common to open and closed class words. For the late components, the amplitude of each TRF for each electrode was averaged within a latency range of 300–600 ms for the open class words and 500–800 ms for the closed class words. Furthermore, the late components were analyzed separately for speech rate, word position, and parts of speech. In the TRFs for the open class words with each speech rate and those for the open class words in each word position the mean amplitudes within a latency range of 300–600 ms were calculated for each feature (i.e., fast, slow, beginning, middle, and end). In order to estimate the word position effect on the late component for the open class words (i.e., the N400), we calculated the differences in amplitude between the beginning, middle, and end positions. In the TRFs for each part of speech, mean amplitudes were calculated within a latency range of 300–600 ms for each open class word (i.e., general/proper noun, verb, adjective, and adverb) and 500–800 ms for each closed class word (i.e., article/determiner, pronoun, BE verb, auxiliary verb, and preposition). The TRFs for numbers, interrogative words, relative pronouns, and conjunctions were excluded from the analysis because we did not find a clear slow negativity in the grand-averaged waveforms.

Similarly to the amplitude analysis, peak latencies of the N1 and P2 were determined for each word class. For the late components, peak latencies were detected separately for speech rate, word position, and parts of speech. To detect peak latencies, we used a dynamic time warping (DTW) algorithm, which is a technique to non-linearly map two temporal sequences that vary in time or speed (Sakoe and Chiba, 1978). DTW has been used to automatically detect peak latencies of ERP components (Assecondi et al., 2009; Zoumpoulaki et al., 2015). First, we detected the peak latencies of grand-averaged TRF waveforms across all participants for each feature. Then, the TRF for each participant and the grand-averaged TRF were mapped onto a common time axis using DTW, so that the time point corresponding to the peak latency obtained from the grand-averaged TRF was automatically identified. The grand-averaged TRFs for the pronouns, BE verbs, and auxiliary verbs had no clear peaks; therefore, the latency for these features were not used for further analysis. We calculated the latency differences of the late component between the beginning, middle, and end positions. A total of 117 EEG features were used for subsequent analyses.

### Correlation Analysis and Score Prediction

Pearson correlation coefficients (*r*) were calculated to assess the relationships between each electrophysiological feature and the EIKEN CSE listening scores. EEG features with | *r*| ≥ 0.2 were used to predict individual listening scores. In cases where | *r*| ≥ 0.2 was observed for multiple electrodes for a feature, we used the electrode with the highest *r*. The EEG features were transformed into z-scores before further analyses. For the prediction model, we trained a least absolute shrinkage and selection operator (LASSO) regression model using the selected EEG features. The loss function of the LASSO is

(2) $$\begin{array}{c} ℒ\left(\beta \right)=\frac{1}{2N}\sum_{i=1}^{N}{\left(y_{i}-\beta_{0}-x_{i}^{T}\beta \right)}^{2}+\lambda \sum_{j=1}^{M}\left|\beta_{j}\right|, \end{array}$$

where *N* is the number of training data, *M* is the number of features, β_0_ and β are regression coefficients, *y*_*i*_ and *x*_*i*_ are the true listening score and EEG feature vector at the *i*th sample, respectively, and λ is a regularization hyperparameter. The model was evaluated using LOOCV. For all 199 samples, one sample was used as test data and the remaining samples were used as training data. The optimal hyperparameter, λ, in the regularization term of LASSO was selected from 500 evenly spaced values ranging between 0 and 5 by performing the 10-fold cross-validation in which λ with the minimum root MSE (RMSE) was selected by the training data. This procedure was performed repeatedly until all samples were applied to the test data. Finally, we calculated Pearson correlation coefficients to evaluate the concordance between the true and predicted listening scores. To estimate the contribution of the features, regression coefficients of each EEG feature were averaged across all models, and their absolute values were normalized in the range of 0 to 1.

### Analysis on the Effect of Age of Acquisition

The age of acquisition (age at which the learner started L2 acquisition) has long been suspected to be a critical factor influencing ERP responses elicited by L2 stimuli, particularly in the domain of syntax (Weber-Fox and Neville, 1996), although it remains unclear how much influence it actually has on the brain activity involved in L2 processing (Caffarra et al., 2015). Therefore, as an additional analysis, we calculated Spearman correlation coefficients to assess the relationships between the age of acquisition and the English listening score and between the age of acquisition and each EEG feature.

## Results

### Task Accuracy and English Listening Scores

The accuracy for the task in the EEG experiment ranged from 0.09 to 1.00 (mean ± SD: 0.73 ± 0.02; median: 0.82). The scores for the English listening test conducted after the EEG experiment (EIKEN CSE listening scores) ranged from 328 to 687 (mean ± SD: 539.1 ± 67.4; median: 551.0). These results indicate that participants ranged widely in English listening proficiency level. We found a high correlation between task accuracy and listening score (Spearman correlation coefficient *ρ* = 0.81; 95% confidence interval: 0.74, 0.87; *p* = 3.23 × 10^–48^).

### Characteristics of the Grand-Averaged TRFs for the Open and Closed Class Words

**FIGURE 3:**
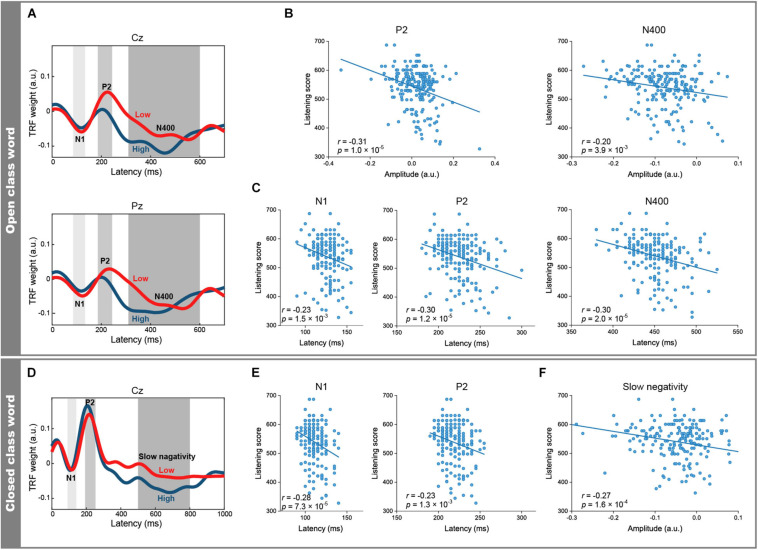
Differences in TRFs between open and closed class words by English proficiency. **(A)** Grand-averaged TRFs for open class words across 49 participants with higher listening scores (blue) and across 51 participants with lower listening scores (red). The peak latencies of the N1, P2, and N400 components (Pz) were longer in the participants with lower scores (125, 230, and 530 ms, respectively) than those with higher scores (120, 200, and 420 ms, respectively). **(B)** The mean P2 and N400 (Cz) amplitudes of the open class words were negatively correlated with the listening scores. **(C)** The N1, P2, and N400 peak latencies (Pz) of the open class words were negatively correlated with the listening scores. **(D)** Grand-averaged TRFs for the closed class words (Cz) across 49 participants with higher listening scores (blue) and across 51 participants with lower listening scores (red). The peak latencies of the N1 and P2 components were longer in the participants with lower scores (115 and 215 ms, respectively) than in those with higher scores (110 and 205 ms, respectively). **(E)** The peak latencies of the N1 and P2 (Cz) of the closed class words were negatively correlated with the listening scores. **(F)** The mean amplitudes of the slow negativity (Cz) of the closed class words were negatively correlated with the listening scores.

The grand-averaged TRFs for the open class words across all 199 participants showed three components that corresponded to the N1, P2, and N400 (Figure 2), all at midline channels. The peak latencies at Cz were 120, 215, and 455 ms, respectively. Similarly, the grand-averaged TRFs for the closed class words showed three components corresponding to N1, P2, and a slow negativity (Figure 2). The peak latencies at Cz were 110, 210, and 700 ms, respectively. To clarify the differences in the neural responses between different levels of English proficiency, we analyzed grand-averaged TRFs for the open and closed class words separately from individuals who showed higher listening scores (*n* = 49; mean score 611 ± 23; CEFR B2 level or higher; Figure 3A) and lower listening scores (*n* = 51; mean scores 446 ± 49; CEFR A1/A2 level; Figure 3D). These waveforms indicated that the temporal characteristics of the responses differed between the two groups. The individuals with lower scores showed longer peak latencies of the N1, P2, and N400 (125, 230, and 530 ms at Pz, respectively) for the open class words compared with those with higher scores (120, 200, and 420 ms, respectively). They also showed longer peak latencies of the N1 and P2 (115 and 215 ms at Cz, respectively) for the closed class words compared with those with higher scores (110 and 205 ms, respectively).

### Correlations Between English Listening Scores and EEG Features

In order to investigate whether the peak latencies and amplitudes of each component were linearly related to the listening scores, we calculated Pearson correlation coefficients. Including the peak latencies of the aforementioned open and closed class words, 17 EEG features of the N1, P2, N400, and slow negativity showed significant correlations (| *r*| > 0.2) with the listening scores (Table 1). The N1 peak latencies of both the open and closed class words were negatively correlated with the listening scores (open class: Pz, *r* = −0.23, *p* = 1.5 × 10^–3^, Figure 3C; closed class: Cz, *r* = −0.28, *p* = 7.3 × 10^–5^, Figure 3E). However, no significant correlation was found between N1 amplitudes and listening scores. Similarly to the N1, P2 peak latencies of the open (Cz, *r* = −0.21, *p* = 2.7 × 10^–3^; Pz, *r* = −0.30, *p* = 1.2 × 10^–5^, Figure 3C) and closed class words (Cz, *r* = −0.23, *p* = 1.3 × 10^–3^, Figure 3E; Pz, *r* = −0.22, *p* = 2.0 × 10^–3^) were negatively correlated with the listening scores. In addition, the P2 amplitudes of the open class words were negatively correlated with the listening scores (Cz, *r* = −0.31, *p* = 1.0 × 10^–5^, Figure 3B).

**TABLE 1:** Electroencephalographical features showing correlation (*| r*| > 0.2) with the listening scores.

Component	Feature		Ch	*r* (95% CI)	*p* values
N1	Open class	lat	Pz	−0.23 (−0.37, −0.07)	1.5 × 10^–3^
	Closed class	lat	Cz	−0.28 (−0.42, −0.13)	7.3 × 10^–5^
P2	Open class	lat	Cz	−0.21 (−0.33, −0.09)	2.7 × 10^–3^
			Pz	−0.30 (−0.43, −0.16)	1.2 × 10^–5^
		amp	Cz	−0.31 (−0.44, −0.15)	1.0 × 10^–5^
	Closed class	lat	Cz	−0.23 (−0.36, −0.09)	1.3 × 10^–3^
			Pz	−0.22 (−0.33, −0.10)	2.0 × 10^–3^
Late component	Open class	lat	Pz	−0.30 (−0.43, −0.17)	2.0 × 10^–5^
		amp	Cz	−0.20 (−0.33, −0.07)	3.9 × 10^–3^
	Closed class	amp	Cz	−0.27 (−0.41, −0.11)	1.6 × 10^–4^
	Fast	lat	Cz	−0.22 (−0.37, −0.06)	1.9 × 10^–3^
	Slow	lat	Cz	−0.24 (−0.36, −0.11)	7.8 × 10^–4^
			Pz	−0.38 (−0.50, −0.26)	3.4 × 10^–8^
	Beginning-end	amp	Pz	−0.21 (−0.36, −0.05)	2.5 × 10^–3^
	General noun	lat	Cz	−0.24 (−0.37, −0.09)	5.8 × 10^–4^
			Pz	−0.33 (−0.46, −0.21)	1.0 × 10^–6^
	Proper noun	lat	Cz	−0.22 (−0.36, −0.06)	2.1 × 10^–3^
	Verb	lat	Pz	−0.21 (−0.33, −0.08)	2.9 × 10^–3^
	Adjective	lat	Cz	−0.21 (−0.34, −0.07)	2.7 × 10^–3^
	Adverb	lat	Fz	−0.23 (−0.34, −0.09)	1.3 × 10^–3^
	BE-verb	amp	Cz	−0.21 (−0.36, −0.06)	3.1 × 10^–3^

*lat, latency; amp, amplitude; and 95% CIs, 95% confidence intervals (lower bound and upper bound).*

**FIGURE 4:**
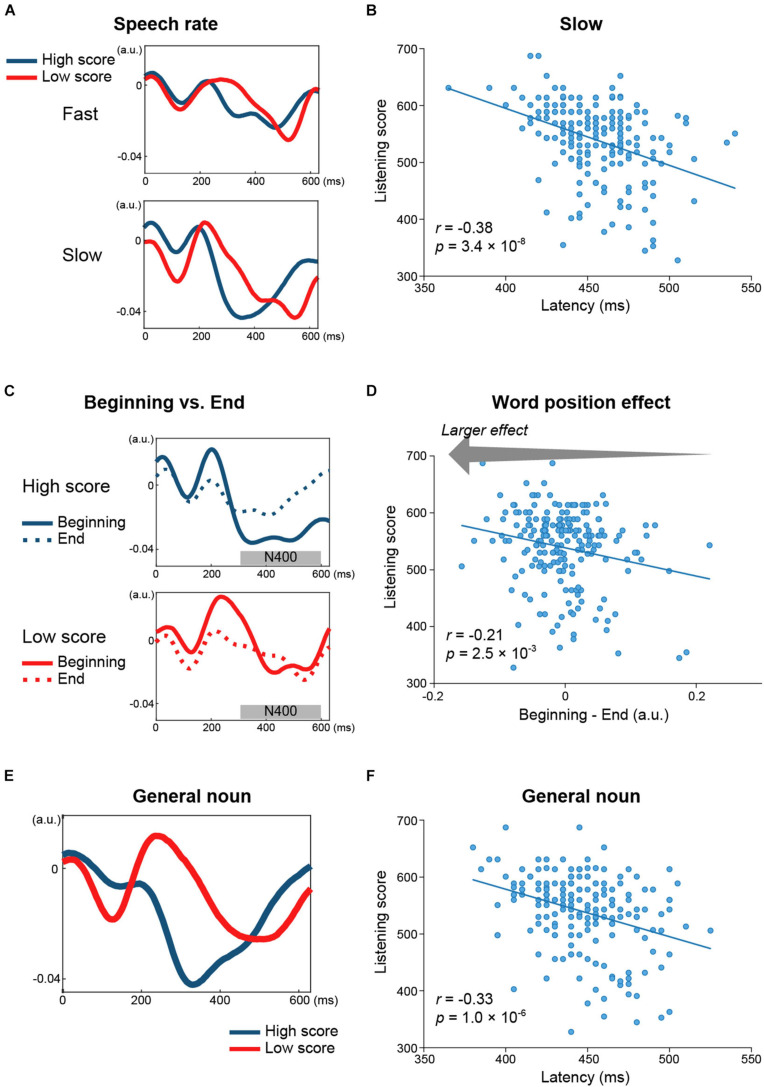
Relationship between the N400 and English proficiency, estimated separately for speech rate, parts of speech, and word position. **(A)** Grand-averaged TRFs (Cz) for words spoken at a fast (up) and slow (bottom) speech rate across 49 participants with higher listening scores (blue) and across 51 participants with lower scores (red). The N400 peak latencies differed between the two groups for both speech rates. **(B)** Negative correlation between N400 peak latencies for slow speech (Pz) and the listening scores. **(C)** Grand-averaged TRFs (Pz) for general nouns in participants with higher (blue) and lower (red) listening scores showing clear between-group differences in N400 peak latencies. **(D)** The N400 peak latencies of general nouns (Pz) were negatively correlated with the listening scores. **(E)** Grand-averaged TRFs (Pz) for words at the beginning (solid line) and end (dashed line) of the speech stimuli in participants with higher (blue) and lower (red) listening scores. The difference in N400 amplitudes between the beginning and end positions of speech stimuli (i.e., the word position effect) was larger in the group with higher listening scores than in the group with lower listening scores. **(F)** The word position effect was negatively correlated with the listening scores. This indicated that the N400 amplitude was lower in the end position than in the beginning position, which was to a larger extent in the participants with higher proficiency.

As for the late component (i.e., N400 for the open class words and the slow negativity for the closed class words), negative correlations with the listening scores were found for the amplitudes of both word classes (open class: Cz, *r* = −0.20, *p* = 3.9 × 10^–3^, Figure 3B; closed class: Cz, *r* = −0.27, *p* = 1.6 × 10^–4^, Figure 3F) and also in the peak latencies of the open class words (Pz, *r* = −0.30, *p* = 2.0 × 10^–5^, Figure 3C). Furthermore, the relationship between the late components and the listening scores were investigated in terms of the speech rate, parts of speech, and word position. For the speech rate, the correlation coefficient was more negative for slow speech (Cz, *r* = −0.24, *p* = 7.8 × 10^–4^; Pz, *r* = −0.38, *p* = 3.4 × 10^–8^) than for fast speech (Cz, *r* = −0.22, *p* = 1.9 × 10^–3^; Figures 4A,B). For parts of speech, the following features showed negative correlations with the listening scores: peak latencies of general nouns (Cz, *r* = −0.24, *p* = 5.8 × 10^–4^; Pz, *r* = −0.33, *p* = 1.0 × 10^–6^, Figures 4C,D), proper nouns (Cz, *r* = −0.22, *p* = 2.1 × 10^–3^), verbs (Pz, *r* = −0.21, *p* = 2.9 × 10^–3^), adjectives (Cz, *r* = −0.21, *p* = 2.7 × 10^–3^), adverbs (Fz, *r* = −0.23, *p* = 1.3 × 10^–3^), and amplitude of BE verbs (Cz, *r* = −0.21, *p* = 3.1 × 10^–3^). For word position, the difference in amplitude between the beginning and end positions in the speech stimuli (i.e., beginning-end) was negatively correlated with the listening scores (Pz, *r* = −0.21, *p* = 2.5 × 10^–3^, Figures 4E,F).

### Predictive Model for English Proficiency Based on EEG Features

**FIGURE 5:**
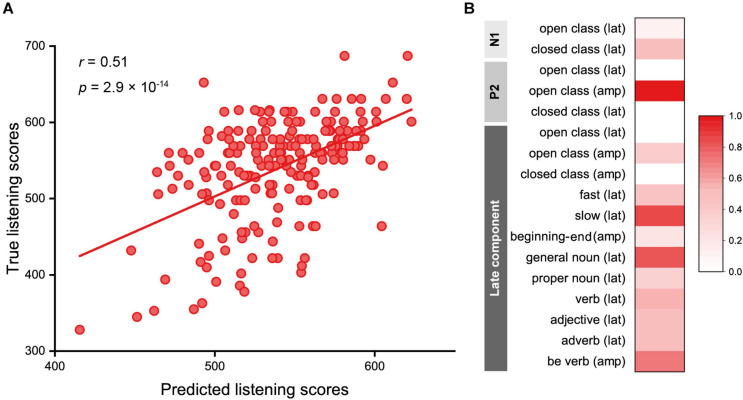
Results of the predictive model for English proficiency based on EEG features. **(A)** The regression model predicted true listening scores, with a mean absolute difference between the true and predicted listening scores of 40.6 ± 36.1. The Pearson correlation coefficient was 0.51 (*p* = 2.9 × 10^–14^). **(B)** Contributions of the EEG features shown in the range of 0 to 1.

Using the 17 EEG features, we predicted listening scores using LASSO regression. The regularization parameter, λ, optimized using 10-fold cross-validation ranged from 0.83 to 2.25, where 12 to 14 features were selected, and the RMSE for the training data ranged from 56.9 to 60.2. The Pearson correlation coefficient between the true and predicted scores of the test data was 0.51 (95% confidence interval: 0.40, 0.60; *p* = 2.9 × 10^–14^), and the mean absolute difference between the true and predicted listening scores was 40.6 ± 36.1 (Figure 5A). The features that showed high contribution to the prediction (>0.5) were as follows (Figure 5B): P2 amplitudes of the open class words (the contribution value: 1.00); peak latencies of the N400 for slow speech (0.86), general nouns (0.83), verbs (0.55), and adjectives (0.50); amplitudes of the slow negativity for BE verbs (0.71); and N1 latencies of the closed class words (0.53).

### Correlations With Age of Acquisition

The age of acquisition did not show any correlation (Spearman correlation coefficient, | *ρ*| < 0.2) with the English listening score or any of the EEG features.

## Discussion

In this study, we measured EEG signals while native Japanese speakers with widely ranging English proficiency levels listened to English natural speech to estimate the TRFs for various types of word features (i.e., word class, word position, speech rate, and parts of speech). Our results showed significant linear correlations between listening scores and EEG responses for several word features. The most important result of our study was that English proficiency for non-native speakers could be predicted on the basis of EEG responses; we observed good concordance between the real and predicted listening scores (correlation coefficient of 0.51). Previous EEG studies in late L2 learners demonstrated differential responses between groups with high and low L2 proficiency by comparing the neural responses of words comprising semantic incongruencies or syntactic violations with those of congruous/correct words (Ojima et al., 2005; Rossi et al., 2006; Newman et al., 2012; Bowden et al., 2013). To the best of our knowledge, there has not been any study that has predicted L2 proficiency using EEG responses. Evidence that L2 proficiency can be estimated using brain activity was demonstrated in a recent functional near-infrared spectroscopy (fNIRS) study. Lei et al. (2020) achieved high accuracy (∼80%) for classifying high and low L2 proficiency by applying machine learning on fNIRS data. In addition to the classification, their study differed from our study in that spatial brain activation information was used. In our study, we used temporal activation information for regression to predict L2 proficiency to take advantage of the high temporal resolution of EEG. Our results demonstrated that L2 proficiency could be predicted from the cortical responses within 1 s (N1, P2, and late components) of the input of each word during continuous speech. The use of EEG signals enabled the direct evaluation of rapid L2 processing in the brain that is not possible with conventional tests.

We found negative correlations between the N1 peak latencies of both word classes and the listening scores. The N1 component is involved in the perception of auditory stimuli and is modulated by selective attention, where amplitudes are higher for attended than unattended stimuli (Hillyard et al., 1973; Hink et al., 1978; Hansen and Hillyard, 1980). The importance of selective attention in L2 learning has been previously shown. In a previous ERP study, Batterink and Neville (2014) showed that L2 learners who spontaneously direct greater attention to open class words rather than closed class words when processing L2 input gain better syntactic learning. In eye-tracking studies on Japanese learners of English, participants who paid more attention to the task-relevant contents showed better task performance, although most participants did not read selectively (Prichard and Atkins, 2018, 2019). In addition, adult bilinguals with high L2 proficiency have shown greater cognitive control as compared with those with low L2 proficiency (Xie, 2018; Xie and Pisano, 2019; Dash and Kar, 2020). These results indicate that more attention to vital points in L2 input leads better comprehension. One of the vital points for speech perception is word onset. It has been proposed that the N1 reflects natural speech segmentation by allocating greater attention to word onsets (Sanders et al., 2002; Sanders and Neville, 2003a; Abla et al., 2008). Sanders and Neville (2003a) reported that the initial syllables of words produce larger N1s than medial syllables when listening to natural speech. The word onset effect was also observed for nonsense words presented as continuous speech after participants had successfully learned these words (Sanders et al., 2002). Given that the N1 could be an index of natural speech segmentation, the shorter N1 latencies in individuals with high proficiency might reflect their faster speech segmentation of natural speech compared with those with low proficiency.

The P2 amplitudes of the open class words and the P2 latencies of both word classes were negatively correlated with the listening scores. Similarly to the N1, the auditory P2 has been found to be modulated by attention: P2 amplitudes decrease as levels of attentiveness increase (Crowley and Colrain, 2004), which is in contrast with the N1 amplitude, and moreover, it increases during the process of falling asleep (Nielsen-Bohlman et al., 1991; Ogilvie et al., 1991; Winter et al., 1995). Therefore, a possible explanation for the smaller P2 amplitude in individuals with higher proficiency is that they allocate greater attention to each word when listening to speech compared with those with lower proficiency. Furthermore, the maintenance of attention would lead to top-down processing by using contextual information. In previous ERP studies on audiovisual speech perception, visual information that predicted an upcoming speech sound had shorter P2 and N1 latencies and lower P2 amplitudes compared with the perception of audio speech alone (van Wassenhove et al., 2005; Ganesh et al., 2014; Sorati and Behne, 2019). Furthermore, temporal facilitation (i.e., shortening of latencies) of the N1 and P2 increased as visual information became more predictive of upcoming auditory targets (van Wassenhove et al., 2005; Ganesh et al., 2014). These results suggest that P2 and N1 latencies and P2 amplitudes will decrease if the information preceding the upcoming word is useful for predicting the word. On the basis of these findings, our results indicate that individuals with higher proficiency construct more contexts while listening to speech to help predict upcoming words, which leads to the accelerated processing of each word.

Similarly to the N1 and P2 latencies, the peak latencies of the late component of the open class words, which corresponds to the N400, showed a negative correlation with the listening scores. The negative correlation was found regardless of speech rate or parts of speech. On the basis of numerous EEG studies that have reported evidence that the N400 reflects semantic processing (Kutas and Federmeier, 2011), our result indicates that semantic processing is faster if English proficiency is higher. Individuals with higher proficiency showed not only shorter N400 latencies but also shorter N1 and P2 latencies. Therefore, we suggest that in those with high proficiency, faster low-order processing, such as phonetic perception and speech segmentation, leads to an acceleration of high-order processing, such as semantic processing. Our result of varied N400 peak latencies dependent on language proficiency is consistent with our previous study (Ojima et al., 2005), where we compared the N400 effect for semantic violations in written English sentences between English native speakers, Japanese native speakers who were highly proficient in English, and Japanese native speakers who had low English proficiency. The N400 latency was longest in the Japanese native speakers with low English proficiency and shortest in the English native speakers, which suggested that the N400 latency could be an index of language proficiency. The present findings further demonstrated that the N400 latency for L2 speech without any violations has a linear correlation with L2 proficiency.

The N400 amplitude of the open class words also showed a negative correlation with the listening scores; that is, the N400 was larger in individuals with higher listening scores. This finding is inconsistent with a previous study. Newman et al. (2012) demonstrated that, in native Spanish speakers who learned English as an L2, N400 amplitudes of semantically congruous words were higher in participants with lower proficiency than in those with higher proficiency, whereas the amplitudes of semantically incongruous words were not influenced by proficiency. This was attributed to the increased cost of semantic integration in the participants with lower proficiency due to less efficient lexical access and/or poorer ability to predict words in well-formed sentences. Our contrasting result might be because of differences in stimulus modality and presentation method. In the previous study, sentences were visually presented one word at a time with a 200-ms interval between words. In contrast, we presented words as part of continuous natural speech, which may introduce greater difficulty in recognizing each word individually, depending on listening proficiency. Our result could be explained from previous studies on speech segmentation. Like the N1, the N400 has also been proposed as an index of online speech segmentation processing (Sanders et al., 2002; Sanders and Neville, 2003b; Abla et al., 2008). Sanders and Neville (2003b) reported that late Japanese learners of English showed a larger N400 to words that were presented as continuous English speech but did not show N1 word onset effects. In another study, low-proficiency learners showed a larger N400 to non-words involved in continuous speech after learning the non-words but showed no N1 word onset effects (Sanders et al., 2002). They proposed that the N1 amplitude may be indexing fast, online speech segmentation in high-proficiency learners and native speakers, whereas the later N400 effect may reflect a slower or more variable segmentation process. Furthermore, numerous studies have shown that the N400 is elicited by meaningful stimuli in various modalities, including written and spoken language and nonverbal stimuli (Kutas and Federmeier, 2011) and that amplitudes vary on the basis of the ease of accessing information from long-term memory and integrating semantic representations into the preceding context (Kutas and Federmeier, 2000). Taken together, our result suggests that individuals with higher proficiency are better able to segment natural speech as a result of extracting more meaningful information from each word.

Previous studies have shown that N400 amplitude can be an index for L2 proficiency, especially in the early learning stage. For example, McLaughlin et al. (2004) showed that, after only 14 h of classroom L2 instruction, N400 amplitude differed for L2 words and pseudo-words. Pu et al. (2016) conducted an experiment with a translation priming paradigm in which L2 words were followed by mother-tongue words (translations of the prime words or not), and showed that the N400 priming effect was presented after less than 4 h of laboratory L2 vocabulary training. In these studies, words were presented one-by-one, and participants were required to perform a judgment task on the words. In the present study, we presented natural speech (conversations and monologs) to the participants and analyzed the EEG signal during their listening. Our finding that the N400 amplitude and latency for words in natural speech had a correlation with the L2 score provides a possibility of assessing L2 proficiency from EEG signals for language input we experience in daily life.

The difference in N400 amplitudes between word positions (i.e., beginning position minus end position) was also negatively correlated with the listening scores. It has been shown that N400 amplitudes of open class words are modulated by the position of the eliciting word within a congruent sentence, where words in later positions produce smaller N400s than those in earlier positions (Kutas et al., 1988; Van Petten and Kutas, 1990). Decreased N400 amplitudes in later positions are caused by the incremental buildup of semantic constraints (Kutas and Federmeier, 2011). Therefore, our result indicates that highly proficient individuals build up and use more contextual information from the beginning of the speech to the end, which enables better comprehension.

The amplitudes for the closed class words, as well as the open class words, were negatively correlated with the listening scores. The analyses for parts of speech revealed a negative correlation between the amplitudes of the BE verbs and the listening scores. Several different usages of BE verbs were grouped into one category in our study; however, previous behavioral research on the acquisition of English as an L2 has shown that different usages of BE verbs are learned at different stages of L2 development. According to the natural order hypothesis (Krashen, 1982), the copula BE (as in “He *is* great”) is acquired earlier than the auxiliary BE (as in “He *is* running”). The correlation between the amplitudes of the BE verbs and the listening scores that we observed may be linked to L2 developmental stages.

Our study has several limitations. For example, we targeted EEG responses to words, based on the above-mentioned previous studies regarding ERPs and L2 proficiency, that is, only EEG responses for words were used in the predictive model for English listening proficiency. One of the major difficulties found in L2 listening comprehension is the difficulty of phonetic perception before word processing, because, in most cases, the L1 and L2 have different phonemes and syllable systems. In addition, difficulties in L2 comprehension at word level inevitably lead to difficulties in comprehension at sentence and discourse levels. Therefore, including EEG features for lower-level (i.e., phonetic perception) and higher-level processing (i.e., sentence and discourse) could create a more accurate prediction model. Further research is needed to clarify this point. The second limitation of our study is that the results did not show how the characteristics of TRFs obtained from the native Japanese speakers differed from those from native speakers of English. This point should be clarified by future work. Third, predicting proficiency directly from the preprocessed EEG data using deep learning methods could make it possible to decrease the efforts for the EEG analysis and also increase the prediction accuracy. In order to conduct this approach, more data samples are needed. Fourth, we did not collect the participants’ educational backgrounds, but they may affect our results. Finally, although the use of EEG signals has advantages to assess language processing, EEG measurement is more expensive and more difficult to conduct than the conventional paper-based tests. For the application of our research, low-cost and easy-to-use EEG devices, which can be used by anyone without the need for special training, are required. Recently, the development of easy-to-use EEG devices has progressed, and, in the future, when low-cost, high-quality, easy-to-use devices are available, our method will be useful in the field of L2 learning.

In conclusion, in this study, we generated a predictive model for Japanese speakers’ English listening proficiency on the basis of EEG signals measured while they were listening to natural English speech. This study provides important information that L2 or foreign language ability can be assessed using neural signatures, without the need for conventional paper tests.

## Data Availability Statement

The data that support the findings of this study are available upon request to the corresponding author.

## Ethics Statement

The studies involving human participants were reviewed and approved by The Ethics Committee for Human and Animal Research of the National Institute of Information and Communications Technology. The participants provided their written informed consent to participate in this study.

## Author Contributions

AM and AI designed the experiments and analyzed the data. KN and SO prepared the speech materials. AM, AI, and JK conducted the experiments. YN, YY, and HW produced techniques for EEG analysis. AI and SO wrote the first draft of the manuscript. All authors revised the draft and approved the final version.

## Conflict of Interest

The authors declare that this study received funding from JSOL Corporation. The funder had the following involvement with the study: discussion about the data.

## Footnotes

1 http://www.eiken.or.jp/eiken/en/grades/

2 https://textinspector.com/

3 https://web.sas.upenn.edu/phonetics-lab/facilities/

4 https://sourceforge.net/projects/wavesurfer/

5 https://www.eiken.or.jp/eiken/en/association/products/; https://www.eiken.or.jp/eiken/en/grades/

6 https://sccn.ucsd.edu/wiki/Artifact_Subspace_Reconstruction_(ASR)
