# Supplementary material for: Prediction of Second Language Proficiency Based on Electroencephalographic Signals Measured While Listening to Natural Speech
Source: Front Hum Neurosci. 2021 Jul 16;15:665809. doi: 10.3389/fnhum.2021.665809 (PMC8322447; doi:10.3389/fnhum.2021.665809)
Supplement: Supplementary file 1 [file Data_Sheet_1.docx]

Supplementary Material

# Supplementary Table 1. Parts of speech analyzed in the present study, original tags in Text Inspector, and example words.

| **Parts of speech** | **Tags by Text Inspector^†^** | **Example words** |
| --- | --- | --- |
| General noun | NN (noun, singular or mass) | woman |
|  | NNS (noun, plural) | characters |
| Proper noun | NP (proper noun, singular) | Kathy |
| Verb | VV (verb, base form) | go |
|  | VVD (verb, past tense) | watched |
|  | VVG (verb, gerund/participle) | wondering |
|  | VVN (verb, past participle) | made |
|  | VVP (verb, present, non-3rd p.) | return |
|  | VVZ (verb, present, 3rd p. sing.) | happens |
| Adjective | JJ (adjective) | interesting |
|  | JJR (adjective, comparative) | better |
|  | JJS (adjective, superlative) | least |
| Adverb | RB (adverb) | often |
|  | RBR (adverb, comparative) | faster |
|  | RBS (adverb, superlative) | earliest |
| Number | CD (cardinal number) | three |
| Article/Determiner | DT (determiner) | each |
|  | DAT (determiner, article) | the |
| Pronoun | EX (existential there) | there |
|  | PN (pronoun neutral) | someone |
|  | PP (personal pronoun) | we |
|  | PP$ (possessive pronoun) | our |
| Interrogative word | PWH (wh-pronoun) | who |
|  | PWS (possessive wh-pronoun) | whose |
|  | WRB (wh-abverb) | when |
| Relative pronoun | THAT (complementizer) | that |
|  | PWH (wh-pronoun) | who |
|  | PWS (possessive wh-pronoun) | whose |
|  | WRB (wh-abverb) | when |
| BE verb | VB (verb be, base form) | be |
|  | VBD (verb be, past) | were |
|  | VBG (verb be, gerund/participle) | being |
|  | VBN (verb be, past participle) | been |
|  | VBZ (verb be, pres., 3rd p. sing.) | is |
|  | VBP (verb be, pres., non-3rd p.) | are |
| Auxiliary verb | MD (modal) | would |
|  | VD (verb do, base form) | do |
|  | VDD (verb do, past) | did |
|  | VDZ (verb do, pres., 3rd p. sing.) | does |
|  | VDP (verb do, pres., non-3rd p.) | do |
|  | VH (verb have, base form) | have |
|  | VHD (verb have, past) | had |
|  | VHZ (verb have, pres. 3rd p. sing) | has |
|  | VHP (verb have, pres. non-3rd p.) | have |
| Preposition | IN (preposition/subord. conj.) | for |
|  | TO (to) | to |
| Conjunction | CO (coordinating conjunction) | but |

†https://textinspector.com/

# Supplementary Table 2. Annotations of parts of speech and phonemes an example sentence shown in Figure 1.

| **Word** | **Parts of Speech (tag by Text Inspector)** | **Parts of speech category** | **Phoneme (ARPABET)** | **Phoneme category** |
| --- | --- | --- | --- | --- |
| He | PP | Pronoun | HH | fricative |
|  |  |  | IY | long vowel |
|  |  |  |  |  |
| gets | VVZ | Verb | G | plosive |
|  |  |  | EH | short vowel |
|  |  |  | T | plosive |
|  |  |  | S | fricative |
|  |  |  |  |  |
| most | PN | Pronoun | M | nasal |
|  |  |  | OW | diphthong |
|  |  |  | S | fricative |
|  |  |  | T | plosive |
|  |  |  |  |  |
| of | IN | Preposition | AH | short vowel |
|  |  |  | V | fricative |
|  |  |  |  |  |
| his | PP$ | Pronoun | HH | fricative |
|  |  |  | IH | short vowel |
|  |  |  | Z | fricative |
|  |  |  |  |  |
| new | JJ | Adjective | N | nasal |
|  |  |  | Y | semivowel |
|  |  |  | UW | long vowel |
|  |  |  |  |  |
| clients | NNS | Noun | K | plosive |
|  |  |  | L | liquid |
|  |  |  | AY |  |
|  |  |  | AH | short vowel |
|  |  |  | N | nasal |
|  |  |  | T | plosive |
|  |  |  | S | fricative |
|  |  |  |  |  |
| through | IN | Preposition | TH | fricative |
|  |  |  | R | liquid |
|  |  |  | UW | long vowel |
|  |  |  |  |  |
| a | DAT | Determiner | AH | short vowel |
|  |  |  |  |  |
| website | NN | Noun | W | semivowel |
|  |  |  | EH | short vowel |
|  |  |  | B | plosive |
|  |  |  | S | fricative |
|  |  |  | AY | diphthong |
|  |  |  | T | plosive |
